# Self-reference Network-Related Interactions During the Process of Cognitive Impairment in the Early Stages of Alzheimer’s Disease

**DOI:** 10.3389/fnagi.2021.666437

**Published:** 2021-03-24

**Authors:** Ping-Hsuan Wei, Haifeng Chen, Qing Ye, Hui Zhao, Yun Xu, Feng Bai, on behalf of Alzheimer’s Disease Neuroimaging Initiative

**Affiliations:** ^1^Department of Neurology, Affiliated Drum Tower Hospital of Medical School and The State Key Laboratory of Pharmaceutical Biotechnology, Institute of Brain Science, Nanjing University, Nanjing, China; ^2^Jiangsu Province Stroke Center for Diagnosis and Therapy, Nanjing, China; ^3^Nanjing Neuropsychiatry Clinic Medical Center, Nanjing, China

**Keywords:** self-reference network, mild cognitive impairment, interaction, modulation, dorsal attention network, salience network

## Abstract

**Background**: Normal establishment of cognition occurs after forming a sensation to stimuli from internal or external cues, in which self-reference processing may be partially involved. However, self-reference processing has been less studied in the Alzheimer’s disease (AD) field within the self-reference network (SRN) and has instead been investigated within the default-mode network (DMN). Differences between these networks have been proven in the last decade, while ultra-early diagnoses have increased. Therefore, investigation of the altered pattern of SRN is significantly important, especially in the early stages of AD.

**Methods**: A total of 65 individuals, including 43 with mild cognitive impairment (MCI) and 22 cognitively normal individuals, participated in this study. The SRN, dorsal attention network (DAN), and salience network (SN) were constructed with resting-state functional magnetic resonance imaging (fMRI), and voxel-based analysis of variance (ANOVA) was used to explore significant regions of network interactions. Finally, the correlation between the network interactions and clinical characteristics was analyzed.

**Results**: We discovered four interactions among the three networks, with the SRN showing different distributions in the left and right hemispheres from the DAN and SN and modulated interactions between them. Group differences in the interactions that were impaired in MCI patients indicated that the degree of damage was most severe in the SRN, least severe in the SN, and intermediate in the DAN. The two SRN-related interactions showed positive effects on the executive and memory performances of MCI patients with no overlap with the clinical assessments performed in this study.

**Conclusion**: This study is the first and primary evidence of SRN interactions related to MCI patients’ functional performance. The influence of the SRN in the ultra-early stages of AD is nonnegligible. There are still many unknowns regarding the contribution of the SRN in AD progression, and we strongly recommend future research in this area.

## Introduction

Alzheimer’s disease (AD) is a neurodegenerative disease accompanied by an irreversible decline in memory, and there is currently no effective treatment (Rafii and Aisen, 2020). Two early stages have been defined that play key roles in AD curative treatment, namely, mild cognitive impairment (MCI) and subjective cognitive decline, in which patients’ network damage is still partially reversible at the neuronal level. A high rate of approximately 10–15% is reported for MCI which annually progresses to AD, and subjective cognitive decline possessing lighter cognitive symptoms is regarded as occurring prior to MCI. Both stages have received much attention in recent years as a possible precursor to this most common dementia state (Cai et al., 2015).

Resting-state functional magnetic resonance imaging (fMRI) has been widely used to investigate the pathogenesis of networks in the course of the disease and has attracted increasing attention. However, little evidence of a self-reference network (SRN) has been found in AD studies. It appears that most research placed the self-reference processing of the SRN under that of default-mode network (DMN) concepts; therefore, there is not much active research being done on their differences (Whitfield-Gabrieli and Ford, 2012; Davey et al., 2016; de Caso et al., 2017; Soch et al., 2017; Kubera et al., 2020). SRN shares some similarities with the DMN in brain regions and the processing function of self-reference (Potvin et al., 2019), whereas the operational type and activated regions (including driving and driven hubs) in the brain have been reported to be different. Wang et al. (2020) has defined the driving hub and driven hub, of which both are composed of brain regions that act similarly in the activation process within a network. The difference between them is that the driving hub takes an active role rather than the passive role taken by the driven hub at the initiation of an activation.

Moreover, neuroimaging has revealed consistent activations in the medial prefrontal cortex (MPFC) and posterior cingulate cortex extending to the precuneus both during explicit self-reference tasks and during rest (Whitfield-Gabrieli et al., 2011). Importantly, the functions between dorsal medial prefrontal cortex (dMPFC) and ventral medial prefrontal cortex (vMPFC) are different (Schwiedrzik et al., 2018; Lieberman et al., 2019). SRN mediates the explicit self-reference in the dMPFC during tasks vs. the DMN actions in the default-mode self-reference in the vMPFC during rest (Whitfield-Gabrieli et al., 2011). The regions mentioned above are major driving hubs within each network. According to the above, the precuneus is involved in all self-reference processing. As mentioned in regard to the driving hubs, the posterior cingulate cortex and precuneus only takes part in the active role within the activation of DMN, while serving a passive role within the activation of SRN (Whitfield-Gabrieli et al., 2011; Wang et al., 2020). Interestingly, dMPFC studies have attracted less attention in AD (Xi et al., 2013; Jedidi et al., 2014; Kurth et al., 2015). Instead, there is more concern with social behavior (Dejean et al., 2016; Goelman et al., 2019; Piva et al., 2019) and psychosis (e.g. depression; Shiota et al., 2017; Schulze et al., 2018) than with neurosis in these studies.

Regarding interactions with other networks, correlations between emotion and attention to cognition scale performance have been clinically discovered, and self-reference processing may be partially involved (Berkovich-Ohana et al., 2012; Amft et al., 2015; Catalino et al., 2020; Tomova et al., 2020; Van der Gucht et al., 2020). The dorsal attention network (DAN) and salience network (SN), which function across both high-level cognitive and attention networks (Arkin et al., 2020; Shi et al., 2020), participate in the regulation of networks between state switching of the brain (Gao and Lin, 2012; He et al., 2014; Chand et al., 2018). Specifically, the right fronto-insular region of the SN plays a critical role in switching between the DMN and the central executive network (He et al., 2014), and the DAN modulates the in-between activity and is damaged in MCI; thus, it is responsible for patients’ cognitive impairment (Chand et al., 2018). The actional patterns in AD progression indicate that the mechanisms of healthy cognition and memory are all based on balance. Ultimately, prior stimulation then forms the necessary sensation to attention, and the normal establishment of those functions comes afterwards (Berger et al., 2015; Qin et al., 2016).

Notably, the relationship of the SRN to the other networks in AD remains unclear. In particular, the SRN effect on cognition is associated with AD. The only closer relationship mentioned in the last decade was the overlap between self-reference processing and salience processing and between self-reference processing and executive control processing regions found in amnestic MCI (Bai et al., 2016), in which the patient’s cognitive performance corresponded to the decoupled functional connection (FC) within and between modules of a network (Contreras et al., 2019) but not age (Sullivan et al., 2019). Nonetheless, it is difficult to show the directly engaged network based on the interpretation in this research. Given that there are many investigations on the DMN rather than the SRN in AD research, there is a crucial need for SRN research. Furthermore, the impact of SRN interactions that contribute to patient cognition in the disease is nonnegligible.

In this study, we aimed to investigate the interaction of the SRN between the DAN and the SN and the relationship of its patterns combined with behavioral and cognitive development in the course of the disease to promote further research on the SRN in AD.

## Materials and Methods

### Participants

A total of 65 subjects, including 43 with MCI and 22 cognitively normal subjects as healthy controls (HC), participated in the study. HC were free of memory complaints (beyond those of normal aging), verified by a study partner. MCI subjects had a subjective memory concern as reported by the subject, study partner, or clinician. All study subjects met the ADNI inclusion and exclusion criteria. In the ADNI, HC are nondepressed, non-MCI, and presented without dementia and have Mini-Mental State Examination (MMSE) scores of 24–30 (inclusive) and a Clinical Dementia Rating (CDR) score of 0. Inclusion criteria for ADNI MCI were MMSE scores of 24–30 (inclusive), a subjective memory concern, a CDR of 0.5, an absence of significant levels of impairment in other cognitive domains, and essentially preserved activities of daily living.

### Neuropsychological Data

The demographic and clinical measures from the ADNI included in this analysis were age; education; sex; and Clinical Dementia Rating Scale: sum of boxes (CDRSB), Alzheimer’s Disease Assessment Scale cognitive subscale (ADAS-Cog11, ADAS-Cog13 and ADAS-Cog Q4), MMSE, Rey Auditory Verbal Learning Test (RAVLT), Logical Memory Test: total number of units recalled (LDELTOTAL), Trail Making Test-B (TRABSCOR), Functional Activities Questionnaire (FAQ), Montreal Cognitive Assessment (MoCA), and Everyday Cognition test: the patient reported version (ECogPT) scores.

### Alzheimer’s Disease Neuroimaging Initiative (ADNI)

The ADNI was launched in 2003 by the National Institute on Aging (NIA), the National Institute of Biomedical Imaging and Bioengineering (NIBIB), the Food and Drug Administration (FDA), private pharmaceutical companies, and nonprofit organizations as a $60 million, 5-year public-private partnership. The primary goal of the ADNI has been to test whether serial magnetic resonance imaging (MRI), functional MRI, other biological markers, and clinical and neuropsychological assessments can be combined to measure the progression of MCI and early AD. The determination of sensitive and specific markers of very early AD progression is intended to aid researchers and clinicians in developing new treatments and monitoring their effectiveness and to lessen the time and cost of clinical trials. To date, the ADNI has three phases, ADNI-1, ADNI-GO, and ADNI-2, consisting of cognitively normal individuals, individuals with MCI, and individuals with dementia or AD. For more information, see http://www.adni-info.org.

### Standard Protocol Approvals, Registrations, and Patient Consent

The ADNI was approved by the institutional review board at each site and was compliant with the Health Insurance Portability and Accountability Act. Written consent was obtained from all participants at each site.

### MRI Acquisition

All subjects were scanned on a 3.0-Tesla MRI scanner (GE Healthcare, Philips Medical Systems). Resting-state functional images were obtained by an echo-planar imaging sequence (EPI: a fast MRI technique that allows the acquisition of single images in as little as 20 ms and the performance of multiple-image studies in as little as 20 s (De LaPaz, 1994) with the following parameters: 140 time points; repetition time (TR) = 3,000 ms; echo time (TE) = 30 ms; flip angle = 80°, number of slices = 48; slice thickness = 3.3 mm spatial resolution = 3 × 3 × 3 mm^3^ and matrix = 64 × 64. All original image files are available to the general scientific community. Detailed descriptions of the resting-state fMRI and MRI scanner protocols are available online^1^. Scan quality was evaluated by the ADNI MRI quality control center at the Mayo Clinic to exclude “failed” scans because of motion, technical problems, or significant clinical abnormalities (e.g., hemispheric infarction).

### Resting-State Functional Image Preprocessing

The fMRI data were processed with the Data Processing Assistant for Resting-State fMRI v2.3 (DPARSFA)^2^ and Resting-State fMRI Data Analysis Toolkit^3^ based on the Statistical Parametric Mapping 12 (SPM12)^4^ and MATLAB (The Math Works, Inc.; Natick, MA, USA) programs (Chao-Gan and Yu-Feng, 2010). The first 10 volumes of the scanning session were abandoned to allow for magnetization equilibration effects. Then, the remaining images were corrected for timing differences in acquisition among slices and head motion effects. No subjects performed a head motion of >3.0 mm of displacement or >3.0° of rotation during the scan. Next, the obtained images were spatially normalized into Montreal Neurological Institute echo-planar imaging templates, resampled to 3 × 3 × 3 mm^3^ voxels, and smoothed with a Gaussian kernel of 6 × 6 × 6 mm^3^ (full width at half-maximum, FWHM). The nuisance signals, including 24 head motion parameters and global mean, white matter, and cerebrospinal fluid signals, were regressed out as covariates of no interest. Finally, the resulting data were bandpass-filtered within the frequency range of 0.01 and 0.08 Hz to reduce the low-frequency drift and high frequency cardiac and physiological respiration noise.

### Resting-State Networks Definition

Seed-based FC analysis was used to construct resting-state networks. The spherical region of interest (ROI) (radius = 8 mm) centered at the dMPFC (Montreal Neurological Institute [MNI] space: −0, 52, 26) (Andrews-Hanna et al., 2010), the medial frontal gyrus (MFG) (MNI space: −8, 57, 12/5, 54, −15) (Jacova et al., 2013), and the bilateral intraparietal sulcus (IPS) (MNI space: −25, −53, 52/25, −57, 52) (Woodward et al., 2011; Ham et al., 2015) served as seed regions for the SRN, bilateral SN, and bilateral DAN, respectively. These seed regions have been widely used to identify the corresponding networks in prior studies. For each subject, an average time series for the ROI was computed as the reference time course. Pearson cross-correlation analysis was then conducted between the average signal change in the dMPFC, MFG, and IPS and the time series of whole-brain voxels. Next, Fisher’s z-transform was used to improve the normality of the correlation coefficients (Lowe et al., 1998). Finally, the individual maps of each network were acquired.

## Statistical Analysis

### Demographic and Neuropsychological Data

The composite scores were applied to enhance statistical reliability by means of reducing random variability and eliminating floor and ceiling effects (Wilson et al., 2010). The *χ*^2^ test was applied in the comparisons of sex. One-way analysis of variance (ANOVA) was applied in the comparisons of education. The Kruskal–Wallis test was applied in age and other neuropsychological data comparisons, with Monte Carlo significance at *p* < 0.05 due to the nonnormal distributions.

### Group-Level Interaction Analysis

Two-way ANOVA with network types (i.e., SRN, left and right DAN, left and right SN) and the two groups (i.e., HC and MCI) was conducted to identify the brain regions showing significant interaction between the two networks in a voxel-wise manner. The thresholds were set at a corrected *p* < −0.05, determined by Monte Carlo simulation for multiple comparisons (AlphaSim-corrected voxel-wise *p* < 0.01, FWHM = 6 mm, cluster size = 756 mm^3^). *Post hoc* analysis was conducted to determine the internetwork differences among the groups. To further investigate the associations between cognitive scores and internetwork differences among the two groups, partial correlation analysis was performed, with age, sex, and education included as covariates. All data were analyzed using SPM12 and SPSS Statistics 22 software (SPSS, Inc., Chicago, IL, USA), with statistically significant differences (*p* < 0.05, Monte Carlo simulation) included.

## Results

### Demographic and Neuropsychological Data

As shown in Table 1, no significant differences in age, years of education, or sex were detected between the groups. In consideration of the main disease effect, MCI subjects displayed significantly worse performance on general cognition than the HC subjects, excluding ECogPT Divided Attention. Notably, the scores of CDRSB, ADASs, FAQ, TRABSCOR, and ECogPTs and two RAVLTs (i.e., the Forgetting and Percent Forgetting) correlated positively with the disease progression or functional damage degree, with a score of 0 corresponding to normal or no impairment and higher scores representing damage severity. The higher score of MMSE, MoCA, LDELTOTAL, and the other RAVLTs (i.e., Immediate Recall and Learning Score) correlated positively with normal performance (Farias et al., 2008; Battista et al., 2017; Moradi et al., 2017).

**Table 1:** Demographic and neuropsychological data.

Items	HC (*n* = 22)	MCI (*n* = 43)	*p*-value^a^
Demographic Data			
Age (years)	73.77 (5.00)	74.22 ± 2.82	0.644
Education (years)	16.27 ± 2.05	15.66 ± 2.53	0.333^b^
Sex (male/female)	7/15	21/22	0.111^c^
Neuropsychological Data			
CDRSB	0.02 (0)	1.66 (2)	<0.001
ADAS11	5.38 (4)	9.88 (7)	<0.001
ADAS13	8.62 (4)	15.93 (11)	<0.001
ADASQ4	2.76 (2)	5.44 (4)	<0.001
MMSE	28.9 (2)	27.8 (3)	0.01
MoCA	25.48 (3)	22.9 (4)	0.001
RAVLT: Immediate recall	47.38 (14)	33.59 (13)	<0.001
RAVLT: Learning	6.29 (4)	3.98 (5)	0.001
RAVLT: Forgetting	3.29 (2)	4.85 (3)	0.024
RAVLT: Percent Forgetting	28.25 (21.47)	63.11 (52.91)	<0.001
LDELTOTAL	14.67 (4)	6.78 (4)	<0.001
TRABSCOR	89.29 (31)	107.85 (74)	0.024
FAQ	0.5 (0)	3.88 (8)	<0.001
ECogPT: Memory	1.6 (0.5)	2.14 (0.94)	0.001
ECogPT: Langue	1.31 (0.44)	1.86 (0.82)	0.002
ECogPT: Visual-spatial	1.17 (0.29)	1.45 (0.79)	0.017
ECogPT: Planning	1.08 (0.2)	1.47 (0.8)	<0.001
ECogPT: Organization	1.17 (0.42)	1.46 (0.67)	0.039
ECogPT: Divided attention	1.52 (0.63)	1.78 (0.75)	0.064
ECogPT: Total score	1.31 (0.29)	1.73 (0.79)	<0.001

*Note: values with normal distributions are presented as the mean ± standard deviation (SD); values with nonnormal distributions are presented as the median (interquartile)*. *χ^2^ test was applied in the comparisons of sex. One-way Kruskal-Wallis test was applied in age and all neuropsychological data comparisons*. *^a^Monte Carlo significant*. *^b^The *p*-value was obtained by one-way ANOVA*. *^c^The *p*-value was obtained by *χ*^2^ test*. *Abbreviations: HC, healthy control; MCI, mild cognitive impairment; CDRSB, Clinical Dementia Rating Scale: sum of boxes; ADAS, Alzheimer’s Disease Assessment Scale cognitive subscales; MMSE, Mini-Mental State Examination; MoCA, Montreal Cognitive Assessment; RAVLT, Rey Auditory Verbal Learning Test; LDELTOTAL, Logical Memory Test: total number of units recalled; TRABSCOR, Trail Making Test-B; FAQ, Functional Activities Questionnaire; ECogPT, Everyday Cognition test: the patient reported version*.

### Identification of Network Interactions

**Figure 1:**
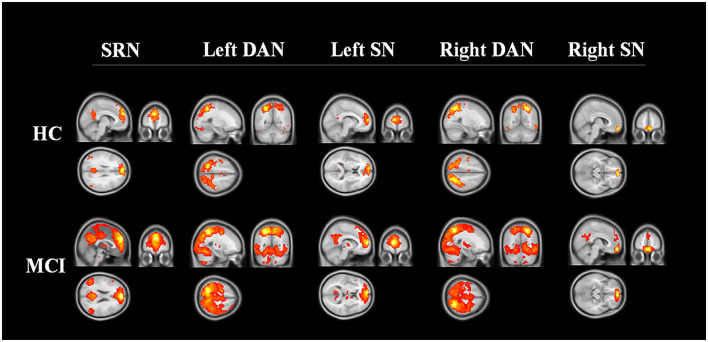
Three networks constructed by region of interest. The networks of HC and MCI showed similar distribution patterns across the majority of the clusters, including the medial frontal, temporal, parietal cortical regions (corrected threshold at *p* < 0.05, Monte Carlo simulation). MCI patients utilized larger regions in all constructed networks than HC. Abbreviations: SRN, self-reference network; DAN, dorsal attention network; SN, salience network; HC, healthy control; MCI, mild cognitive impairment.

The spatial maps of each reconstructed network are shown in Figure 1. A qualitative visual inspection of networks between the two groups showed similar patterns, in which distributions were demonstrated across the majority of the clusters, including diffuse subcortical and cortical sites, with a corrected threshold at *p* < 0.05 (Monte Carlo simulation), for example, the SRN in medial frontal and other cortical middle regions; the DAN in temporal and parietal regions; and the SN in frontal cortical regions. Nevertheless, MCI patients utilized larger regions in all constructed networks than the HC.

**Figure 2:**
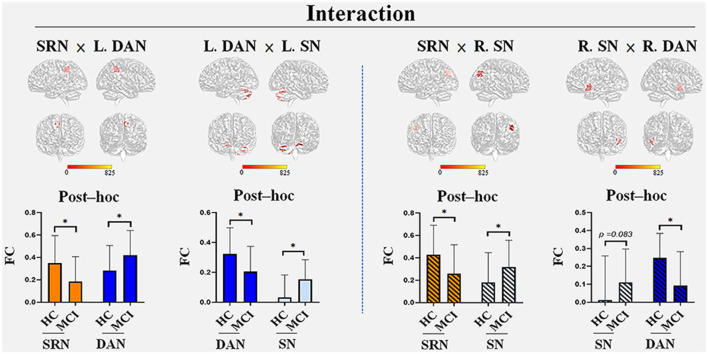
The interactions among the three networks with group differences. Four interactions were found: the SRN demonstrated interactions with the DAN and SN in the left and right hemispheres, respectively, whereas the DAN and SN demonstrated interactions in both hemispheres. (1) The SRN and left DAN demonstrated interactions in the main regions of the right precuneus; (2) the left DAN and left SN demonstrated interactions in both the left and right cerebellum regions, including the posterior lobe, inferior lobe, superior lobe, pyramis, and declive; (3) the SRN and right SN demonstrated interactions in the main region of the right angular gyrus; (4) the right DAN and right SN demonstrated interactions in the left superior temporal gyrus. The brain regions that were demonstrated above were not limited to the defined ROI coordinates (i.e., the left or right hemisphere) of each constructed network due to the networks’ known whole-brain distribution. *Post hoc* tests showed the internetwork differences among the groups: (1) the SRN showed a decrease in FC in all its related interactions, whereas increases in FC were found in the corresponding networks (the left DAN and right SN) within the interaction with the SRN; (2) the decreased FC in the DAN and increased FC in the SN were demonstrated within their own interactions of both hemispheres in MCI patients compared with HC. All interactions were significant (corrected *p* < 0.05, Monte Carlo simulation) between HC and MCI patients, except that of the right SN (*p* = 0.083) with the right DAN. Abbreviations: L, left; R, right; SRN, self-reference network; DAN, dorsal attention network; SN, salience network; FC, functional connectivity; HC, healthy control; MCI, mild cognitive impairment; *Monte Carlo significant.

We found four interactions between each pair of networks of the SRN, DAN, and SN, the details of which are shown in Table 2 and Figure 2. The SRN demonstrated interactions with the DAN and SN, respectively, in the left and right hemisphere, whereas the DAN and SN demonstrated interactions in both hemispheres: (1) the SRN and left DAN showed interactions in the main regions of the right precuneus; (2) the left DAN and left SN showed interactions mainly in the left and right cerebellum regions, including the posterior lobe, the inferior lobe, the superior lobe, pyramis, and declive; (3) the SRN and right SN showed interactions in the main region of the right angular gyrus; and (4) the right DAN and right SN showed interactions mainly in the left superior temporal gyrus. The brain regions with the interactions demonstrated above were not limited to the defined ROI coordinates (i.e., the left or right hemisphere) of each constructed network due to the networks’ known whole-brain distribution. Surprisingly, modulations of the SRN through its communication with the left DAN (and right SN) to the interactions of the DAN and SN in the left (and right) hemisphere occurred; however, these modulations happened to be damaged in individuals with MCI.

**Table 2:** Regions showing self-reference network (SRN) interactions with the dorsal attention network (DAN) and salience network (SN).

Interactions	Brain regions	BA	Peak MNI coordinates			Peak intensity	Number of cluster voxels (mm^3^)
			*x*	*y*	*z*		
SRN × Left DAN	Precuneus. R	7/31	12	−42	42	13.7827	864
Left DAN × Left SN	Cerebellum posterior lobe. L Cerebellum inferior lobe. L Pyramis. L	-	−27	−72	−39	11.0646	1458
	Cerebellum posterior lobe. R Cerebellum superior lobe. R Declive. R	-	15	−66	−21	11.4859	1080
SRN × Right SN	Angular gyrus. R	39	48	−72	33	9.5474	810
Right SN × Right DAN	Superior temporal gyrus. L	13/22/38	−45	9	−9	14.8657	756

*Note: BA, Brodmann area; MNI, Montreal Neurological Institute; R, Right; L, Left; *p* < 0.01, AlphaSim corrected*.

*Post hoc* tests showed the internetwork differences among the groups. The SRN showed a decrease in all of its related interactions, whereas the corresponding networks within those networks were all increased in MCI patients compared with HC. The FC of each network within the interaction between the DAN and SN demonstrated a decrease in the DAN and an increase in the SN in both the left and right hemispheres. All interactions were significant (corrected *p* < 0.05, Monte Carlo simulation) between HC and individuals with MCI, except the SN within the interactions between the right DAN and right SN (*p* = 0.083).

### Behavioral Significance of Network Interactions

**Figure 3:**
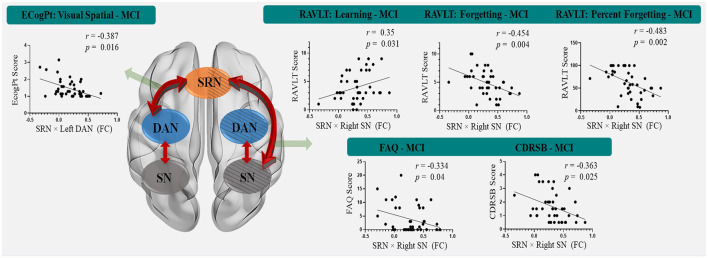
The correlation between SRN-related interactions and cognitive functions in mild cognitive impairment patients. The interaction of the SRN with the left DAN was positively correlated with MCI patients’ visual-spatial performance in the ECogPT test. The interaction of the SRN with the right SN was negatively correlated with the MCI patients’ clinical test scores on the FAQ, CDRSB, and RAVLTs (the Forgetting and the Percent Forgetting), whereas only the RAVLT: Learning Score was positively correlated with the interaction. Notably, the scores of the CDRSB, FAQ, ECogPTs, and two RAVLTs (i.e., the Forgetting and the Percent Forgetting) were positively correlated with the disease progression or functional damage degree, while a higher RAVLT: Learning Score was positively correlated with normal performance. Therefore, all correlations indicating that these functional performance scores of MCI patients are positively correlated with FC were within SRN-related interactions. Red arrows show the interaction patterns among the SRN, DAN, and SN. Green arrows show the significant correlations of the SRN-related interactions to clinical assessments. The background with the oblique line shows networks in the right hemisphere; the background without the oblique line shows networks in the left hemisphere. Abbreviations: MCI, mild cognitive impairment; SRN, self-reference network; DAN, dorsal attention network; SN, salience network; FC, functional connectivity; ECogPT, Everyday Cognition test: the patient reported version; RAVLT, Rey Auditory Verbal Learning Test; FAQ, Functional Activities Questionnaire; CDRSB, Clinical Dementia Rating Scale: sum of boxes.

The significant results of the behavioral significance of SRN-related interactions that correlated only with MCI (no correlation with HC) are presented in Figure 3. The interaction of the SRN with the left DAN correlated positively with MCI patients’ visual-spatial performance in the ECogPT test (*r* = −0.387, *p* = 0.016). The interaction of the SRN with the right SN correlated negatively with the MCI patients’ clinical test scores on the RAVLTs, including Forgetting (*r* = −0.454, *p* = 0.004) and Percent Forgetting (*r* = −0.483, *p* = 0.002); the FAQ (*r* = −0.334, *p* = 0.04), and the CDRSB (*r* = −0.363, *p* = 0.025), whereas only the RAVLT: Learning Scores (*r* = 0.35, *p* = 0.031) were positively related to the interaction due to its assessment design. According to the above results (“Demographic and Neuropsychological Data” section), the higher the scores were on the RAVLT: Learning test regarding the score design, the better the related performance of patients was; in contrast, higher scores on the other assessments were associated with worse functions. All these data indicated a positive relationship between the functional performances of MCI patients and SRN-related interactions.

## Discussion

### First Evidence of SRN Modulations and Its Special Distribution Among the Other Networks

We discovered the interactions among the three networks and brain regions. Four interactions (Table 2 and Figure 2) were not limited to the defined ROI coordinate (i.e., the left or right hemisphere) of each constructed network due to the networks’ known whole-brain distributions. The network normally interacts between hemispheres; therefore, it might be the crossing recruitment within network in order to adapting to the damage functions (Ptak et al., 2020). Furthermore, SRN anatomical structure is located in the midline of cortex. The ROI coordinate of SRN we selected lies in middle area of brain. Accordingly, these cross-hemisphere results shown in SRN-related interactions is actually reasonable.

To emphasize, the SRN showed a fundamental difference from the DMN in its relationship to the DAN and SN concerning both self-referencing and attentional processes. The DMN tends to be passively regulated by both the DAN and the SN, whereas the SRN plays an active role in the relationship. For the SRN and DMN, a lower FC between these networks has been proven to lead to global decline in episodic memory retrieval or the recognition of amnestic MCI (Bai et al., 2012a). Nonetheless, selective changes within the SRN at least preserved the partial task function of amnestic MCI (Bai et al., 2016). Most importantly, the modulation of the SRN to the interaction between the DAN and the SN (hereafter, DAN-SN) was first evidenced in our study. The SRN regulates the DAN-SN in the left hemisphere through its interactions with the DAN and regulates the DAN-SN in the right hemisphere through the SN.

Furthermore, a different distribution of the SRN interacting with only the left DAN and only the right SN in the left and right hemispheres, respectively, vs. the DAN and the SN interacting in both hemispheres in this study, showed the special characteristic of the SRN in its connection with the two networks. This may be related to the laterality. For instance, the significance of network functional lateralization in AD progression is as follows: (1) in the SN, in which right lateralization has been proven (Zhang et al., 2019), the occurrence of connections with the SRN on the right side rather than the left side significantly reduced FC, especially in the right prefrontal cortex, and has been observed in subjective cognitive decline patients (Hu et al., 2017); (2) however, in the DAN, damage patterns (Zhang et al., 2015) and inhibition in the temporal region of the whole brain have been observed in MCI patients (Chand et al., 2018; Zhang et al., 2019), but evidence for lateralization remains debatable (Corbetta and Shulman, 2002; Vossel et al., 2012; Mayrhofer et al., 2019); and (3) in addition, the DMN also presented left lateralization but functional decline with age and AD (Banks et al., 2018), showing insufficient activation in the right prefrontal region but overactivation in the left prefrontal region during memory maintenance and reasoning tasks in MCI patients (Melrose et al., 2018). Nevertheless, the hyperactivation in the DAN and SN and the hypoactivation in the DMN were regarded as compensatory due to damage that had been confirmed to be directly related to the AD pathology in the right hemisphere (Wu et al., 2011; Li et al., 2012). Tau protein accumulation is positively related to neurorehabilitation or neural plasticity, regardless of neuron metabolism or nutrition, in AD (Cope et al., 2018), and beta-amyloid appears to be positively correlated with high neuronal activity (Bero et al., 2011; Mormino et al., 2011). Consequently, AD pathology preferentially occurring in the right hemisphere may be related to the fact that the right hemisphere is dominant in most human brains. Therefore, we believe that network lateralization is a natural balance of the brain and affects SRN distributions. Although lateralization does not affect FC performance, in which the rearrangement mechanism follows different pathological stages in AD progression (Bai et al., 2016; Banks et al., 2018), lateralization may participate in the adaptation or compensatory performance of each network.

### SRN Exhibits Damage at the Early Stage of the Disease

The larger region of all constructed networks shown in MCI patients compared with HC indicates the impact of the disease on the network modules, in which tropology mainly serves a network function (Contreras et al., 2019). Moreover, group differences in the discovered interaction represent differences not only in damage patterns but also in adaptation to AD. For MCI patients, it was shown that the SRN decreased its participation in all its relating interactions, whereas the corresponding networks all increased their participation within those interactions. For the interaction between the DAN and the SN, the FC of the DAN decreased and that of the SN increased, as shown in both the left and right hemispheres. Accordingly, the degree of impairment among the three networks in MCI patients was most severe in the SRN, least severe in the SN, and intermediate in the DAN. This result is similar to previous studies that have proven functional damage in the DAN and SN (Li et al., 2012; Zhan et al., 2016; Bi et al., 2018; Chand et al., 2018) but observed only several compensatory patterns in the SN (Balthazar et al., 2014; He et al., 2014). Another task state study found that the DMN was capable of better reorganization than the SRN in MCI patients with worse memory performance (Bai et al., 2012b, 2016). Moreover, damage in SRN regions (the left triangular part of inferior frontal gyrus) has been reported to be a problem in maintaining longitudinal memory (Bi et al., 2018). Based on the above, we thought that the SRN also suffered more serious damage than other networks, as in the DMN, in which FC alterations within and to other networks have been suggested to be directly related to AD pathology (Ferreira et al., 2019) at the early stage of the disease (Bai et al., 2016; Melrose et al., 2018). The SRN may have less damage adaptation as it has higher specificity but smaller functionality than the DMN (Whitfield-Gabrieli et al., 2011; Bai et al., 2012a, 2016).

### SRN Functional Relationship With Multiple Functions in MCI

The interactional performance of the SRN with the DAN in the left hemisphere was related to only the ECogPT: Visual-spatial score, whereas that of the SRN with the SN in the right hemisphere was related to FAQ, CDRSB, and RAVLTs (including RAVLT: Learning, Forgetting and Percent Forgetting) scores in MCI patients, showing the functional differences between these SRN interactions.

Visual-spatial organization is reported as a fundamental coding principle to structure the communication between distant brain regions (Knapen, 2021). The connection between the cognitive network and basal ganglia network, which processes the primary integration of information, has been proven to be positively related to visual-spatial performance (Bagarinao et al., 2019; Hucke et al., 2020). Moreover, the attention function is known to be closely connected to the visual system (Sharafeldin et al., 2020; Speed et al., 2020). In addition to engaging in the cognition process, networks composed of frontotemporal regions function to integrate multisensory information, and parietal regions manage attention and visual-spatial functions. Accordingly, we suggest that it might be the possible mechanism underlying the effect of the SRN and left DAN interactions related to the visual-spatial performance of MCI patients.

Researchers have previously identified the influences of other networks on AD and MCI patients’ visual-spatial symptoms (Li et al., 2012; Brissenden et al., 2016; Buckley et al., 2017), yet no related study has evaluated the SRN. Our research has provided the first evidence that the visual-spatial performance in MCI patients is affected by the interaction between the SRN and the left DAN.

Next, the SRN also showed an effect on executive and memory function within its interaction with the SN in addition to its own self-referencing. The greater the interaction between the SRN and the right SN is, the more normal the FAQ, CDRSB, and RAVLT performances in MCI patients. Self-reference processing was required more from the SRN than from the DMN when the brain was in a task state and was reflected in the FAQ performance, which is a self-administered functional assessment (Battista et al., 2017) requiring more self-reference processing than other testing scales in this study. These results show that SRN influences are as important as DMN influences on clinical scale scores. Moreover, the CDRSB involves partial executive and memory function assessment, and RAVLTs are tests for episodic memory functions (Battista et al., 2017). The effect we found of a corresponding interaction of the SRN with the SN showed positive enhancement of both the executive and memory functions of MCI patients, especially with no discovery of any two-way impact that occurred in the DMN (showing both positive and negative influences to the cognitions that function different but in the same category (Berger et al., 2015; Gardini et al., 2015; Bi et al., 2018; Melrose et al., 2018) on memory performance (e.g., the RAVLTs used in the study).

Notably, these functions were decreased in MCI patients compared to HC. Although the DAN and SN similarly increased FC within SRN-related interactions, their participation in the regulation of networks between brain-state switching as a feedback loop influenced both themselves and the SRN (Gao and Lin, 2012; He et al., 2014; Chand et al., 2018; Sullivan et al., 2019), indicating a complex explanation of their compensatory effects within SRN-related interactions. In addition, a memory encoding failure is much more likely to occur when the connections of self-reference processing (involved in the SRN and the DMN) are switching between task and rest states of the brain (Bai et al., 2016) while the patient is undergoing clinical assessment. Accordingly, we propose that the relationship between these functions and interactions is highly related to the SRN compensatory ability within the related interaction, which has also been reported to be damaged and therefore does not last long enough to maintain or improve functional performance (Bi et al., 2018).

## Limitations

Since the primary research of this study focuses on the SRN and cognition in AD, less emphasis is placed on neuropsychological assessment considering the self-reference processing function. We should further supplement the related scales and demonstrate a better exploration of SRN development in the course of Alzheimer’s disease at follow-up. Regarding reproducibility, another independent sample should be recruited to confirm the present findings. Therefore, these data should be interpreted with caution.

## Conclusion

We found special regulation of the SRN in cognitive function, with a particular distribution trend between the other networks, the DAN and SN, arranged in both cognitive and attention network systems. The two SRN-related interactions improved some cognitive performance in MCI patients. The fact that no overlap was observed between neuropsychological assessments reflects the different participations of SRN-related interactions. We also demonstrated the damage adaptation among the three networks and pointed out more differences between the SRN and the DMN. On the basis of this primary research on interactions between the SRN and both the DAN and the SN in AD, we strongly suggest that future research should consider the influence of the SRN on cognition. In particular, research conducted in the ultra-early stages may be of more benefit to the field of the disease.

## Data Availability Statement

The raw data supporting the conclusions of this article will be made available by the authors, without undue reservation.

## Ethics Statement

The studies involving human participants were approved by the institutional review board at each site and were compliant with the Health Insurance Portability and Accountability Act. ADNI data are disseminated by the Laboratory for Neuro Imaging at the University of Southern California. The ADNI was performed in accordance with the Good Clinical Practice guidelines, US 21CFR Part 50-Protection of Human Subjects, and Part 56-Institutional Review Boards (IRBs)/Research Good Clinical Practice guidelines Institutional Review Boards (IRBs)/Research Ethics Boards (REBs). More information is available at: http://adni.loni.usc.edu. The patients/participants provided their written informed consent to participate in this study.

## Author Contributions

P-HW: conceptualization, formal analysis, investigation, writing—original draft, and visualization. HC: methodology and software. QY: methodology. HZ: visualization. YX: visualization. FB: supervision. Alzheimer’s Disease Neuroimaging Initiative: resources. All authors contributed to the article and approved the submitted version.

## Conflict of Interest

The authors declare that the research was conducted in the absence of any commercial or financial relationships that could be construed as a potential conflict of interest.

## Footnotes

^1^http://www.adni.loni.usc.edu/methods/documents/mriprotocols/

^2^http://www.rfmri.org/DPARSFA

^3^http://www.restfmri.net

^4^http://www.fil.ion.ucl.ac.uk/spm
